# The mitochondrial genome of *Chelonus formosanus* (Hymenoptera: Braconidae) with novel gene orders and phylogenetic implications

**DOI:** 10.1002/arch.21870

**Published:** 2022-01-28

**Authors:** Rui‐Zhong Yuan, Jin‐Jin Zhou, Xiao‐Han Shu, Xi‐Qian Ye, Pu Tang, Xue‐Xin Chen

**Affiliations:** ^1^ State Key Lab of Rice Biology Zhejiang University Hangzhou China; ^2^ Institute of Insect Sciences, College of Agriculture and Biotechnology Zhejiang University Hangzhou China; ^3^ Hainan Institute Zhejiang University Sanya China; ^4^ Ministry of Agriculture Key Lab of Molecular Biology of Crop Pathogens and Insects Zhejiang University Hangzhou China; ^5^ Zhejiang Provincial Key Laboratory of Biology of Crop Pathogens and Insects Zhejiang University Hangzhou China

**Keywords:** Cheloninae, *Chelonus formosanus*, mitochondrial genome

## Abstract

*Chelonus formosanus* Sonan is an important egg‐larval parasitoid of noctuid moths and a potential candidate for understanding interactions between host and parasitoid mediated by polydnavirues (PDVs). We sequenced and annotated the mitochondrial genome of *C. formosanus*, which is 15,466 bp in length and possesses 38 mitochondrial genes. However, unlike most animal mitochondrial genomes, it contains one extra *trnF* gene. There are five transfer RNA (tRNA) rearrangement events compared with the ancestral gene order, which is a novel rearrangement type in Hymenoptera for all published mitogenomes so far. Phylogenetic trees supported *C. formosanus* from the subfamily Cheloninae was closely related to the subfamily Cardiochilinae and Microgastrinae.

## Graphical abstract

This study obtained the complete mitochondrial genome of *Chelonus formosanus*, provided a thorough description of its genome features and constructed phylogenetic relationships among major Braconidae lineages.

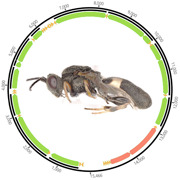

## 1 INTRODUCTION

Cheloninae is a moderate‐sized subfamily of Braconidae with approximately 1523 described species worldwide. Members of the chelonines are solitary koinobiont endoparasitoids of Lepidoptera. *Chelonus formosanus* Sonan (Hymenoptera: Braconidae: Cheloninae) is distributed in Neotropical and Oriental regions, which attacks several lepidopteran species, especially noctuid moths, including *Spodoptera frugiperda*, *S. exigua*, *S. litura*, *Helicoverpa armigera*, and *Mythimna loreyi*, which was thought to be an excellent biological control agent for *Spodoptera* spp. (Tang et al., 2019). Moreover, Cheloninae can possess polydnavirues (PDVs) which is a kind of special insect viruses which shares a symbiotic and mutualistic relationship with some hymenopteran endoparasitoid (Strand et al., 2012). Therefore, *C. formosanus* is a potential candidate for understanding interactions between host and parasitoid mediated by PDVs.

Mitochondrial genome performs an important function in phylogenetic construction, and it is genetically matrilineal inheritance and nonrecombinant with other mitochondrial lineages in insect (Cameron et al., 2007). Gene arrangements are usually conserved within major lineages (Boore, 1999), but may be highly rearranged in certain groups (Cameron et al., 2007; Covacin et al., 2006; Dowton & Austin, 1999; Shao & Barker, 2003; Shao, Campbell & Schmidt, et al., 2001; Shao, Campbell, & Barker, 2001). However, in hymenopteran mitogenomes, frequent gene rearrangements have been identified from broad examinations of gene segments (Dowton & Austin, 1999; Dowton et al., 2003) and whole genome sequences (Cameron et al., 2008; Castro et al., 2006; Cha et al., 2007; Crozier & Crozier, 1993), providing both challenges and opportunities for detecting rearrangement synapomorphies. Other extraordinary features of the hymenopteran mitochondrial genome include the most compositional biased mitogenomes within insects and extremely variable substitution rates between lineages, making the analysis of hymenopteran relationships with mitogenome phylogenomics challenging (Cameron, 2014). Here, we obtained the complete mitochondrial genome of *C. formosanus*, which provided a thorough description of its genome features and constructed phylogenetic relationships among major Braconidae lineages.

## 2 MATERIAL AND METHODS

### 2.1 Species identification and DNA extraction

The specimens of *C. formosanus* were collected from Hainan (109.19 N, 18.37E), Sanya, China in Yacheng in November 2020. The collected specimen was stored in 100% ethanol and stored at −80°C before DNA extraction. Voucher specimen was identified based on the morphology. The genomic DNA was extracted using FastPure Cell/Tissue DNA Isolation Mini Kit (Vazyme Biotech Co., Ltd) according to the manufacturer's instructions. The voucher specimen was kept in the Parasitic Hymenoptera Collection of Institute of Insect Sciences, Zhejiang University.

### 2.2 Next‐generation sequencing and genome assembly

The library was constructed by VAHTS™ Universal DNA Library Prep Kit for Illumina® v9.1, and sequenced by Illumina HiSeq sequencer (150 bp pared‐end) of Novogene. FastQC was used to check the quality of the data, and Trimmomatic was used to trim adaptors and indexes with default parameters (Bolger et al., 2014; Wingett & Andrews, 2018). The target mitochondrial reads were filtered out using BLAST (BLASTn with *E* value: 1 × 10^−5^) against a reference data set containing Braconidae mitochondrial genomes via the FastqExtract script (Crampton‐Platt et al., 2015). The mitochondrial genome was assembled by IDBA‐UD (Peng et al., 2012) and SPAdes version 3.13.1 (Bankevich et al., 2012) with default parameters.

### 2.3 Mitochondrial genome annotation and analysis

Annotation of assembled genome was performed by using MITOS Web Server (Bernt et al., 2013). Start and stop codons of protein‐coding genes (PCGs) were manually adjusted in Geneious Prime v11 by referencing to the published mito‐genomes of Braconidae. The online tRNAscan‐SE service (http://lowelab.ucsc.edu/tRNAscan-SE/) was used to confirm locations of transfer RNA (tRNA) genes. Characteristics of mitochondrial genomes of sequenced species in Braconidae were analyzed, including gene arrangements, base compositions, codon usages, and evolutionary rates (Ka, Ks, and Ka/Ks) of PCGs. Gene rearrangements were analyzed by comparing with the putative ancestral type of *Drosophila melanogaster*. The base composition was obtained by MEGA v7.0 (Tamura et al., 2013). The AT‐skew and GC‐skew were calculated according to formulae: AT‐skew = (A% − T%)/(A% + T%) and GC‐skew = (G% − C%)/(G% + C%) (Perna et al., 1995). The relative synonymous codon usage (RSCU) of all PCGs was calculated in Geneious Prime v11. Synonymous (Ks) and non‐synonymous (Ka) substitution rates of PCGs were calculated using DnaSP v6.12.03 (Rozas et al., 2017).

### 2.4 Phylogenetic analysis

Mitochondrial genomes of 12 species from the family Braconidae were chosen to construct phylogenetic relationship of Braconidae. *Diadegma semiclausum* and *Hyposoter* sp. both from Ichneumonidae were chosen as outgroup. The PCGs were aligned using G‐INS‐i algorithms implemented in MAFFT v7.464, respectively (Katoh & Standley, 2013). The best partition schemes of substitution models for the matrix were searched in PartitionFinder v2 with model selection=BIC and Branch lengths=unlinked between different subsets (Lanfear et al., 2012). Mrbayes v3.2.7a (Ronquist & Huelsenbeck, 2003) and RAxML‐HPC2 v8.2.12 (Stamatakis, 2014) were used to reconstruct the phylogenetic tree, respectively, based on nucleotides (NU) sequences of 13 PCGs. For Bayesian inference analysis (BI), Four simultaneous Markov chains were run for 10 million generations, with tree sampling occurring every 1000 generations, and a burn‐in of 25% of the trees in Mrbayes to phylogenetic analysis. In maximum likelihood (ML) analysis, the GTRGAMMA model and 200 runs for different individual partitions to construct ML trees with 1000 bootstrap replicates.

## 3 RESULTS AND DISCUSSION

### 3.1 Genome structure and organization

A total of 5.08 Gb raw data and 4.86 Gb filtered clean data were produced in this sequencing, with 95.67% of effective rate and only 0.03% of error rate were monitored. The complete mitochondrial genome of *C. formosanus* is 15,466 bp in length (GenBank accession MZ169618), containing 13 PCGs, 23 tRNA genes, and two ribosomal RNA (rRNA) genes. 38 mitochondrial genes were identified in *C. formosanus*. However, unlike most animal mitochondrial genomes, it contains one extra *trnF* gene that can be detected using either the IDBA or SPAdes assemblers. The overlapping nucleotides from six adjacent genes in the mitochondrial genome of *C. formosanus* were discovered to up to 18 bp in total, as the maximum length of overlap were 7 bp in length, locating at one junction (*nad4*‐*nad4l*) and the minimum length of overlap were 1 bp in length, locating one junctions (*trnK‐nad5*). The total intergenic nucleotides were 361 bp in length and dispersed between 17 adjacent genes, ranging from the smallest gene spacer, 1 bp in length locating at one junctions (*trnY*‐*cox1*) to the longest gene spacer, 72 bp in length dispersing between *nad1* and *trnL1*. Neither overlapping nor space nucleotides were found between other 14 adjacent genes.

### 3.2 Base composition and codon usage

The nucleotides composition of the mitochondrial genome of *C. formosanus* has been analyzed, including AT%, GC%, AT skew, and GC skew in different region. In general, the mitochondrial genome performs a significant bias toward A/T in nucleotide composition (Cameron, 2014). The overall nucleotide composition was 40.0% of A, 7.8% of G, 46.6% of T, and 75.5% of C, with an A + T content of 86.6%. *rrnL* has a length of 1171 bp, with an A + T content of 89.4%. *rrnS* has a length of 707 bp, with an A + T content of 89.8%. Since there is no standard way to define the exact boundaries of rRNAs, the criteria of reducing intergenic spacer and overlapping region could not be applied to assign the start codon. Both *rrnL* and *rrnS* conform to the secondary structure models proposed for these genes from other insects. Relative high A + T content in mitochondrial genome is not unusual in Hymenoptera (Oliveira et al., 2008). In the mitochondrial genome of *C. formosanus*, the value of GC skew in almost all genes were positive, and the value of AT skew in most genes were negative, positive AT skew locating at four genes (0.11 in *nad1*, 0.08 in *nad4*, 0.10 in *nad4l*, and 0.05 in *nad5*).

The RSCUs in the mitochondrial genomes of *C. formosanus* displayed an overwhelming preference toward the usage of A and T, especially at the third position, owing to genetic code degeneracies, which to some extent explained the high bias of A + T content in the mitochondrial genome (Table 1). The total number of codons in *C. formosanus* mitochondrial genome is 3721. Among the available codons, there are five most widely used amino acid with its corresponding codons in the following order, Leu‐UUA, Val‐GUU, Ser‐UCA, Ser‐UCU, and Pro‐CCU, respectively, which is consistent with those of other hymenopteran mitogenomes (Song et al., 2016; Wei, Shi, Sharkey, et al., 2010; Wei, Tang, et al., 2010; Wei et al., 2014; Xiao et al., 2011).

**Table 1:** Codon usage in the mitochondrial genome of *Chelonus formosanus*

Amino acid	Codon	Number	RSCU	Amino acid	Codon	Number	RSCU
Ala	GCU	26	2	Arg	CGU	21	2
	GCC	1	0.08		CGC	2	0.19
	GCA	25	1.92		CGA	16	1.52
	GCG	0	0		CGG	3	0.29
Cys	UGU	30	1.88	Ser	AGU	33	0.95
	UGC	2	0.13		AGC	0	0
Asp	GAU	62	1.91		AGA	59	1.69
	GAC	3	0.09		AGG	5	0.14
Glu	GAA	59	1.76		UCU	83	2.38
	GAG	8	0.24		UCC	6	0.17
Phe	UUU	425	1.94		UCA	92	2.64
	UUC	13	0.06		UCG	1	0.03
Gly	GGU	54	1.6	Thr	ACU	50	1.94
	GGC	2	0.06		ACC	3	0.12
	GGA	64	1.9		ACA	48	1.86
	GGG	15	0.44		ACG	2	0.08
His	CAU	52	1.93	Val	GUU	82	2.78
	CAC	2	0.07		GUC	0	0
Ile	AUU	439	1.96		GUA	32	1.08
	AUC	10	0.04		GUG	4	0.14
Lys	AAA	156	1.93	Trp	UGA	68	1.86
	AAG	6	0.07		UGG	5	0.14
Leu	UUA	559	5.41	Tyr	UAU	203	1.95
	UUG	38	0.37		UAC	5	0.05
	CUU	12	0.12	Pro	CCU	45	2.09
	CUC	1	0.01		CCC	1	0.05
	CUA	10	0.1		CCA	39	1.81
	CUG	0	0		CCG	1	0.05
Met	AUA	366	1.84	Gln	CAA	35	1.89
	AUG	31	0.16		CAG	2	0.11
Asn	AAU	292	1.92				
	AAC	12	0.08				

Abbreviation: RSCU, relative synonymous codon usage.

### 3.3 PCGs

**Figure 1:**
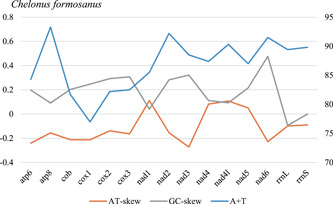
Base composition of protein‐coding genes and two rRNAs in the mitochondrial genome of *Chelonus formosanus*. rRNA, ribosomal RNA

All 13 typical PCGs of mitochondrial genome of *C. formosanus* were successfully detected. The total length of these 13 PCGs was 11,163 bp, comprising 72.18% of the entire mitochondrial genome length. The size of the PCGs in the *C. formosanus* mitochondrial genome is similar to their corresponding orthologs in other insects (Li et al., 2016; Wei, Shi, Sharkey, et al., 2010; Wei, Tang, et al., 2010). The entire A + T content in the *C. formosanus* mitochondrial genome was 86.84%, which ranged from 78.00% (*cox1*) to 92.0% (*atp8*) (Figure 1). The genes with the highest A + T content in the hymenopteran mitochondrial genome are usually *nad6* or *atp8* (Wei et al., 2014). In *C. formosanus*, the A + T content of *atp8* is 92.0%, and the A + T content of *nad6* is 91.6%.

All PCGs in the *C. formosanus* mitochondrial genome started with typical ATN codon (five with ATT, four with ATA, three with ATG, and one with ATC), as those of other Hymenoptera insects (Cameron et al., 2008; Castro & Dowton, 2005; Cha et al., 2007; Crozier & Crozier, 1993; Oliveira et al., 2008; Wei et al., 2009). Approximately all PCGs in *C. formosanus* mitochondrial genome terminated with a complete codon. Only one PCGs (*nad4*) use incomplete stop codons T, which is commonly reported in other invertebrates. The RSCU values show a biased use of A and T nucleotides in *C. formosanus*.

### 3.4 tRNA genes

**Figure 2:**
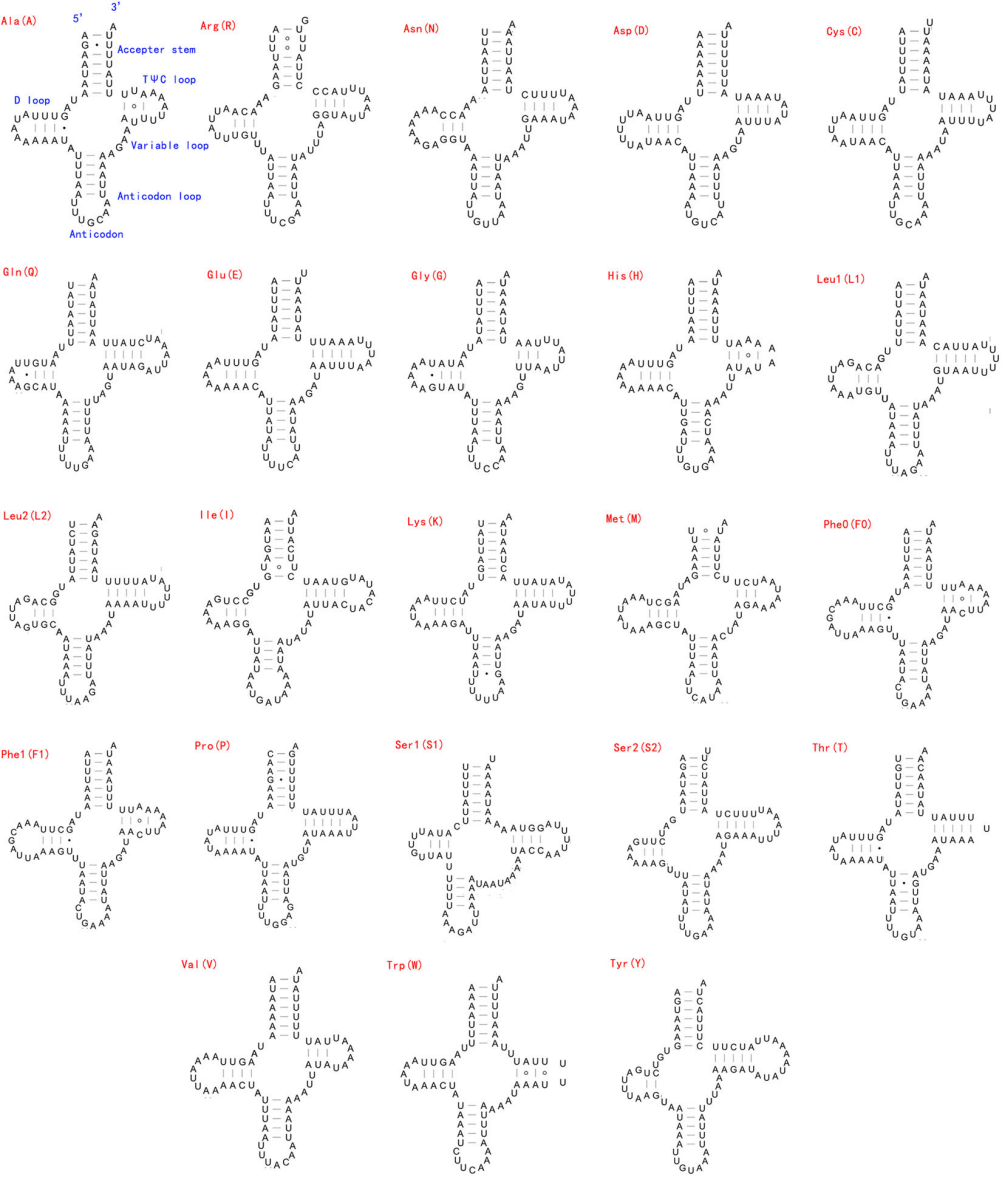
Predicted secondary structures for the 23 typical tRNA genes of *Chelonus formosanus* mitochondrial genome. Base‐pairing is indicated as follows: Watson–Crick pairs by lines, wobble GU pairs by dots and other noncanonical pairs by circles. tRNA, transfer RNA

The entire 22 ancestral tRNAs commonly existing in animal mitochondrial genome were successfully detected in the mitochondrial genome of *C. formosanus*, but unlike other animal mitochondrial genomes, there was an extra *trnF* detected in the mitochondrial genome of *C. formosanus*. These tRNA genes were scattered throughout the mitochondrial genome. For the predicted tRNA, 12 tRNAs encoded in the majority‐coding strand, while the other 11 tRNAs were positioned in the minority‐coding strand. Generally, the length of tRNAs ranges from 62 (*trnH*) to 71 bp (*trnY*). All tRNA genes in the mitochondrial genome of *C. formosanus* have typical clover‐leaf secondary structures (Figure 2). The DHU‐stem (4–9 bp) and TΨC‐stem (1–10 bp) of the mitochondrial tRNA in *C. formosanus* varied in length, while the size of its anticodon loop of all tRNA was 7 bp, except for that in *trnI* (9 bp), and the size of its amino‐acid acceptor stem of all tRNA was 7 bp. Such featured variations are normal in other insect mitochondrial tRNA (Mao et al., 2014; Flook et al., 1995; Li et al., 2016). In the mitochondrial tRNA secondary structures of *C. formosanus*, a total of 21 mismatched base pairs were detected with 11 G‐U pairs, 6 U‐U pairs, 2 A‐A pairs, and 2 A‐C pairs. The number of mismatches is relatively high in the *C. formosanus* mitochondrial tRNAs compared with other insects (Kim et al., 2005).

### 3.5 Gene rearrangement

**Figure 3:**
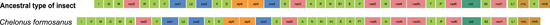
Gene rearrangement in the mitochondrial genome of *Chelonus formosanus*

Rearrangement of tRNA genes is common in the hymenopteran mitochondrial genome, especially those in tRNA clusters, such as in the junctions of AT region‐*nad2*, *nad2*‐*cox1*, *cox2*‐*atp8*, and *nad3*‐*nad5*. Compared with the putative ancestral type of *Drosophila melanogaster*, the mitochondrial genome of *C. formosanus* is similar to other hymenopteran mitochondrial genomes that have high‐frequency rearrangements in tRNA. There are five tRNA rearrangement events found in the three gene clusters (*I‐V‐M‐C‐W‐Q*, *atp6*‐*atp8*, *A‐N‐S1‐F0‐E‐R‐F1*), compared with the ancestral gene order (Figure 3). The rearrangement type in the *C. formosanus* mitochondrial genome is novel in Hymenoptera.

Gene rearrangement events have been usually divided into four categories: translocation, local inversion (inverted in the local position), gene shuffling (local translocation), and remote inversion (translocated and inverted) (Dowton et al., 2002). Five tRNA genes are rearranged, which are remote inversions of *trnV*, *trnC*, *trnW*, and *trnR*, and gene shuffling of *atp8*. In the *C. formosanus* mitochondrial genome, remote inversion was found to be the dominant gene rearrangement event, and the plausible explanation of the local inversions in hymenopteran mitochondrial genomes may be affected by the recombination in the local inversions.

### 3.6 Phylogenetic analyses

**Figure 4:**
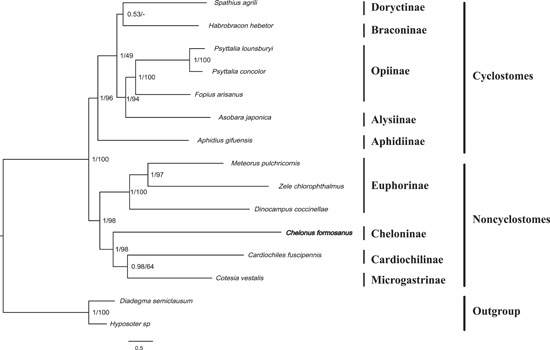
Phylogenetic tree of the tested Braconidae and two outgroups based on 13 PCGs in mitochondrial genomes. The numbers at the nodes separated by “/” indicates the Bayesian posterior probabilities and bootstrap values. PCG, protein‐coding gene

To validate the phylogenetic position of *C. formosanus* within Braconidae, in this study the phylogenetic analysis was constructed based on 13 PCGs by ML and Bayesian method with other 14 insects, including 12 species across the family Braconidae and 2 species in family Ichneumonidae as outgroup. In this study, the phylogenetic analyses based on mitochondrial genomes, recovered the well‐accepted major lineages within the family, and supported the division of Braconidae into Cyclostomes and Noncyclostomes as commonly accepted (Figure 4) (Quicke & van Achterberg, 1990; Sharanowski et al., 2011; Wei, Shi, Chen, et al., 2010). Within the Cyclostomes, Aphidiinae was recovered as a sister clade to the remaining species from Cyclostomes. Meanwhile, consistent with previous studies, within the Noncyclostomes, Euphorinae was recovered as a sister clade to the remaining Noncyclostomes, and *C. formosanus* from the subfamily Cheloninae was closely related to *Cotesia vestalis* and *Cardiochiles fuscipennis*, which belonged to the subfamily Microgastrinae and Cardiochilinae, respectively, with a strong confidence bootstraps value and Bayesian posterior probabilities, and divided with other lineages (Li et al., 2016). Since several subfamilies were not included in our analysis, the phylogenetic relationships within Braconidae should be studied with more mitogenomes.

## CONFLICT OF INTERESTS

The authors declare that there are no conflict of interests.

## AUTHOR CONTRIBUTIONS

**Rui‐Zhong Yuan**: Data curation (lead), Writing – original draft (equal). **Jin‐Jin Zhou**: Resources (equal), Software (equal). **Xiao‐Han Shu**: Data curation‐Supporting, Resources (equal). **Xi‐Qian Ye**: Funding acquisition (equal), Software (equal). **Pu Tang**: Funding acquisition (equal), Project administration (equal), Supervision (equal), Writing – review & editing (lead). **Xue‐Xin Chen**: Funding acquisition (equal), Project administration (equal), Writing – review & editing‐Supporting.

## Supporting information

Supplementary information.
