# Supplementary material for: The mitochondrial genome of Chelonus formosanus (Hymenoptera: Braconidae) with novel gene orders and phylogenetic implications
Source: Arch Insect Biochem Physiol. 2022 Jan 28;111(1):e21870. doi: 10.1002/arch.21870 (PMC9539690; doi:10.1002/arch.21870)
Supplement: Supplementary file 1 — Supplementary information. [file ARCH-111-e21870-s001.docx]

**Table S1**. The mitochondrial genome of all species accessed in NCBI

| Species | Family | Subamily | Accession number |
| --- | --- | --- | --- |
| *Asobara japonica* | Braconidae | Alysiinae | NC_045903 |
| *Aphidius gifuensis* | Braconidae | Aphidiinae | NC_054223 |
| *Habrobracon hebetor* | Braconidae | Braconinae | MN842279 |
| *Cardiochiles fuscipennis* | Braconidae | Cardiochilinae | KF385870 |
| *Spathius agrili* | Braconidae | Doryctinae | NC_014278 |
| *Dinocampus coccinellae* | Braconidae | Euphorinae | MG253265 |
| *Meteorus pulchricornis* | Braconidae | Euphorinae | NC_053259 |
| *Zele chlorophthalmus* | Braconidae | Euphorinae | NC_039181 |
| *Cotesia vestalis* | Braconidae | Microgastrinae | NC_014272 |
| *Fopius arisanus* | Braconidae | Opiinae | MZ128286 |
| *Psyttalia concolor* | Braconidae | Opiinae | MW279212 |
| *Psyttalia lounsburyi* | Braconidae | Opiinae | MW279214 |
| *Hyposoter sp.* | Ichneumonidae | Campopleginae | MG923499 |
| *Diadegma semiclausum* | Ichneumonidae | Campopleginae | EU871947 |
